# Supplementary material for: μeV electron spectromicroscopy using free-space light
Source: Nat Commun. 2023 Jul 24;14:4442. doi: 10.1038/s41467-023-39979-0 (PMC10366080; doi:10.1038/s41467-023-39979-0)
Supplement: Supplementary file 1 — Supplementary information [file 41467_2023_39979_MOESM1_ESM.pdf]

μeV electron spectromicroscopy using free-space light

– SUPPLEMENTARY INFORMATION –

Yves Auad,[1] Eduardo J. C. Dias,[2] Marcel Tencé,[1] Jean-Denis Blazit,[1]
Xiaoyan Li,[1] Luiz Fernando Zagonel,[3] Odile Stéphan,[1] Luiz H. G. Tizei,[1]
F. Javier García de Abajo,[2,4,*] and Mathieu Kociak[1,†]

[1] Université Paris-Saclay, CNRS, Laboratoire de Physique des Solides, 91405, Orsay, France
[2] ICFO-Institut de Ciencies Fotoniques, The Barcelona Institute of Science and Technology, 08860 Castelldefels (Barcelona), Spain
[3] Applied Physics Department, "Gleb Wataghin" Institute of Physics, University of Campinas – UNICAMP, 13083-859 Campinas, SP, Brazil
[4] ICREA-Institució Catalana de Recerca i Estudis Avançats, Passeig Lluís Companys 23, 08010 Barcelona, Spain

Contents

S1. Experimental setup for continuous-wave EEGS spectroscopy S1
A. Light detection S1
B. Laser source and laser injection parameters S2
C. Details on the optical setup used to perform EEGS experiments S3
D. Complementary measurement for the high-Q microresonator S4

S2. Analytical theory for the EEGS probability S4
A. Spherical specimen under plane wave illumination S5
B. Illumination by means of a parabolic mirror S6
C. EEGS under illumination by a parabolic mirror S8

S3. Mie modes in large dielectric spheres S9

S4. EEGS in a large silica sphere S10

S5. On the question of spectral resolution and signal strength in EELS, CL, and EEGS S16

References S17

S1. EXPERIMENTAL SETUP FOR CONTINUOUS-WAVE EEGS SPECTROSCOPY

A. Light detection

For the CL experiments, light detection was performed with an Attolight Mönch system fitted with a ≈ 0.5 numerical aperture. The light collected through the mirror was focused into a single-

*Electronic address: javier.garciadeabajo@nanophotonics.es
†Electronic address: mathieu.kociak@universite-paris-saclay.fr

step-index multimode fiber of 100 μm in diameter (model FG105LVA Thorlabs Inc.) coupled to an optical spectrometer of similar numerical aperture, roughly 0.1. The fiber transmission efficiency was more than 95% within the entire spectral range used throughout this work.

B. Laser source and laser injection parameters

For light injection, a dye laser beam (Pyromethane 597 organic solution, with peak efficiency at 585 nm) was focused on the sample by means of a high-numerical-aperture parabolic reflector after being spatially filtered by a single-mode optical fiber. The focused light beam, of approximately ~ 1 μm in diameter (see Sec. S2 B for a numerical analysis), could be precisely positioned at the edge of a whispering-gallery mode resonator (WGMR; a large dielectric sphere) thanks to a Mönch three-dimensional stage with an ultimate 50 nm precision. The pulsed dye laser operated at a repetition rate of 10 kHz and delivered pulses with a duration in the 20–30 ns range. In contrast to conventional PINEM experiments in ultrafast electron microscopes, the electron and photon temporal synchronization was carried out using a fast blanker placed immediately before the detector, as shown in Fig. 1 in the main text and discussed elsewhere in more detail [1]. The nanosecond-resolved beam blanker selected electrons that crossed the sample during the laser pulse duration, with the beam blanker time window fixed at 250 ns to accommodate a slight variation of the laser pulse delay on the pump laser current. Notice that only a tenth of the transmitted electrons was exposed to the laser action. Typical time-averaged input powers were in the range of

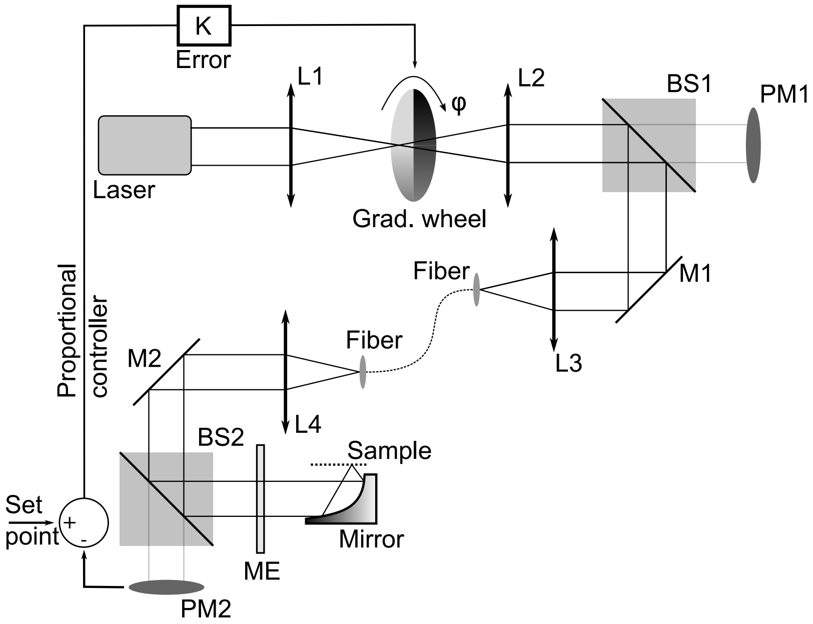

FIG. S1: **Scheme of the optical components used in our EEGS experiment.** The laser output is focused on a gradient wheel density filter. The telescopic lenses L1 and L2 produce an output with unity magnification. The beam splitter BS1 collects 10% of the incident power in the power meter PM1. Finally, the laser beam is focused on the optical fiber. The other side of the output fiber is coupled to L4. M2 and BS2 send the light beam to the parabolic mirror, focusing the beam on the sample plane (dashed line). The boundary between the atmosphere and vacuum (inside the microscope) is represented by the microscope edge ME. L1, L2: achromatic lenses; L3, L4: achromatic aspheric lenses; BS1, BS2: 90:10 beamsplitter; M1, M2: silver mirror; PM1, PM2: power meters.

1–5 mW, thus resulting in 10^{8-9} W/m^2 light intensities. The acquisition time per EELS spectrum in the wavelength series was typically 100 ms. Over this time interval, a set of 5–25 measurements were performed (and subsequently averaged) per wavelength. A direct electron detector (Merlin, from Quantum Detectors) was used, which improved the quantum efficiency and the signal-to-noise ratio of the acquired data. The laser pulse duty cycle (i.e., the product of its pulse length and its repetition rate) was 2×10^{-4}, implying that the average current available for generating an EEGS signal was typically 2×10^{-16} A.

C. Details on the optical setup used to perform EEGS experiments

The elements of the EEGS experimental setup are shown in Fig. S1. The optical design consists of two main parts: the coupling of the dye-laser light beam into the optical fiber (Fig. S1a) and the other side of the optical fiber attached to the microscope light injection (or collection) system (Fig. S1b). The achromatic lenses L1 and L2 in Fig. S1a, which have the same focal point and, thus, form a telescopic imaging system with unity magnification, are used to produce an optical cross-over in which a continuous neutral density gradient wheel is placed. The latter is linked to a servo motor by a mechanical belt and two homemade 3D-printed rotating shafts. Additionally, an Arduino UNO is used to control the servo motor through the standard microcontroller library. The beamsplitter cube BS1 reflects 90% of the incident light. The remaining 10% is sent to the power meter PM1 (Thorlabs S120C sensor with Thorlabs PM100USB interface). The PM1 element is mainly used to determine the transmission percentage across the optical fiber, which is helpful during alignment

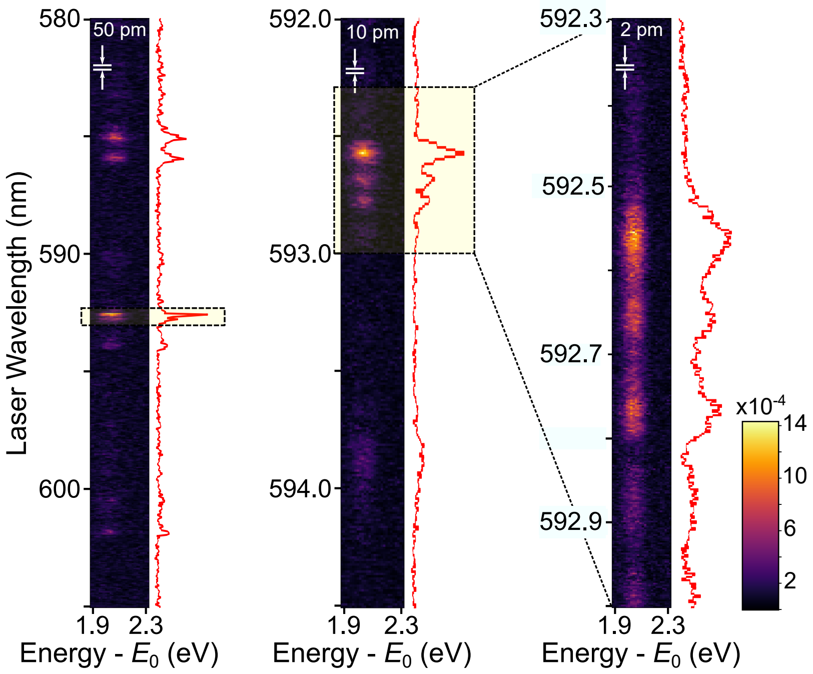

FIG. S2: **Additional series of measurements showing a high-quality factor in a large polystyrene sphere**. We show an intermediate wavelength series, from 592.0 to 594.5 nm, taken with steps of 10 pm. This figure directly relates to Figure 4 in the main text and corroborates the reproducibility of the experimental procedure.

and allows us to estimate the reminiscent chromatic aberration of the optical system. The silver mirror M1 and the achromatic aspheric lens L3 focus the laser beam onto the fiber. In M1, two angular degrees of freedom are controlled by piezoelectric actuators (Thorlabs PIAK10), while L3 is placed on a lens mount with 5 degrees of freedom (3 spatial and 2 angulars). The fiber ends at a standard fiber SMA connector and is mounted on a 3-axis Thorlabs NanoMax^{TM} flexure stage using manual differential micrometers. Alignment is mainly carried out by playing with M1 and the fiber 3-axis stage. For a single-mode fiber, typical transmission values are $\sim$ 4-10% across the Pyrromethene dye range (566–611 nm wavelength), although the gradient-wheel-based control system properly corrects these values. To estimate the focusing power of the parabolic mirror, the field of view of the single-mode optical fiber is directly measured by rastering a < 100 nm diamond crystal containing multiple nitrogen-vacancy (NV) centers, which are known to efficiently emit light in the wavelength range of the dye laser used, thus reducing the effect of chromatic aberration.

D. Complementary measurement for the high-Q microresonator

Related to the measurements shown in Figure 4 in the main text, we plot in Figure S2 an additional wavelength series acquired with 10 pm steps, corroborating the reproducibility of the experimental procedure.

S2. ANALYTICAL THEORY FOR THE EEGS PROBABILITY

The interaction of a swift electron with a spherical object has previously been studied using analytical theory methods to calculate the EELS and cathodoluminescence (CL) emission probabilities [2], which should actually coincide when absorption by the probed materials is negligible (e.g., for the spheres considered in the present work). We derive below an analytical expression for the EEGS probability due to the interaction with an illuminated sphere by relating it to the CL far-field amplitude via the reciprocity theorem.

Considering a specimen subject to external monochromatic illumination of frequency ω, the effect of the optical field on an electron moving with constant velocity v along the z direction is encapsulated in the coupling parameter [3]

$$\beta_{\rm EEGS}(\omega) = \frac{e}{\hbar\omega}\int_{-\infty}^{\infty} dz\, E_z(x_e, y_e, z)\, {\rm e}^{-{\rm i}\omega z/v}, \tag{S1}$$

where (x_e, y_e) defines the electron beam (e-beam) position in the transverse plane, while the amplitude $\mathbf{E}(\mathbf{r})$ is taken such that the time-dependent electric field reads $2{\rm Re}\big\{\mathbf{E}(\mathbf{r}){\rm e}^{-{\rm i}\omega t}\big\}$. We explicitly indicate the frequency dependence of $\beta_{\rm EEGS}(\omega)$ because this is a key ingredient in EEGS. After interaction, the incident zero-loss peak ($\ell = 0$) splits into a series of peaks separated by multiples of the photon energy $\ell\hbar\omega$, with associated probabilities $P_\ell(\omega) = J_\ell^2(2|\beta_{\rm EEGS}(\omega)|)$ satisfying $\sum_{\ell=-\infty}^{\infty} P_\ell(\omega) = 1$. In the present study, the coupling is relatively weak ($|\beta_{\rm EEGS}(\omega)| \ll 1$), and therefore, only the first gain ($\ell = 1$) and loss ($\ell = -1$) peaks are experimentally studied in the transmitted electron spectra, both of them having the same probability $J_1^2\big[2|\beta_{\rm EEGS}(\omega)|\big] \approx |\beta_{\rm EEGS}(\omega)|^2$.

Rather than calculating $\beta_{\rm EEGS}(\omega)$ by first obtaining the total (incident+scattered) field $\mathbf{E}$ and then integrating Eq. (S1), we exploit the reciprocity theorem to relate $\beta_{\rm EEGS}(\omega)$ to the CL far-field amplitude $\mathbf{f}^{\rm CL}(\omega)$ as [3]

$$\beta_{\rm EEGS}(\omega) = \frac{{\rm i}c^2}{\hbar\omega^2}\mathbf{f}^{\rm CL}_{\hat{\mathbf{r}}}(\omega)\cdot\mathbf{E}^{\rm ext}, \tag{S2}$$

where $\mathbf{E}^{\rm ext}$ is the EEGS incident electric field amplitude in the absence of the specimen. In this expression, $\mathbf{f}^{\rm CL}(\omega)$ needs to be computed after reversing the electron velocity vector relative to

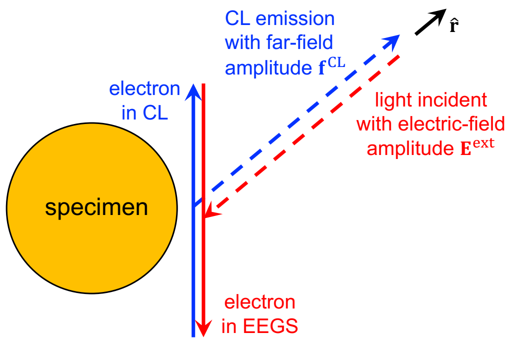

FIG. S3: **Sketch of the geometry involved in the application of the reciprocity theorem.** We relate the EEGS coupling coefficient $\beta_{\rm EEGS}(\omega)$ (red) to the CL far-field amplitude (blue) by means of Eq. (S2). The propagation directions of electron and light are both reversed in CL relative to EEGS.

EEGS, and with the CL emission direction pointing to the EEGS light source, as indicated in Fig. S3. In particular, the CL emission direction is $\hat{\mathbf{r}}$, but the propagation direction of the EEGS incident light is $-\hat{\mathbf{r}}$ (see Fig. S3).

A. Spherical specimen under plane wave illumination

We now take a previously derived analytical expression for the CL far-field amplitude of a sphere in vacuum [4]:

$$\mathbf{f}^{\rm CL}_{\hat{\mathbf{r}}}(\omega) = \frac{c}{\omega}\sum_{l=1}^{\infty}\sum_{m=-l}^{l}\left[t_l^M\psi_{lm}^{M,\rm ext}\,\vec{\zeta}_{lm}(\hat{\mathbf{r}})\,+t_l^E\psi_{lm}^{E,\rm ext}\,\hat{\mathbf{r}}\times\vec{\zeta}_{lm}(\hat{\mathbf{r}})\right], \tag{S3}$$

where l and m run over multipolar components, t_l^ν are the m-independent Mie scattering coefficients for electric (ν =E) and magnetic (ν =M) polarization, and $\psi_{lm}^{\nu,\rm ext}$ are expansion coefficients that express the external evanescent field carried by the electron as a sum over multipoles around the sphere center. In Eq. (S3), we use the vector spherical harmonics

$$\vec{\zeta}_{lm}(\hat{\mathbf{r}}) = \frac{1}{2}\left[C_{lm}^{-}Y_{lm-1}(\hat{\mathbf{r}})+C_{lm}^{+}Y_{lm+1}(\hat{\mathbf{r}})\right]\hat{\mathbf{x}}\,+\frac{\rm i}{2}\left[C_{lm}^{-}Y_{lm-1}(\hat{\mathbf{r}})-C_{lm}^{+}Y_{lm+1}(\hat{\mathbf{r}})\right]\hat{\mathbf{y}}\,+mY_{lm}(\hat{\mathbf{r}})\,\hat{\mathbf{z}}, \tag{S4}$$

where $C_{lm}^{\pm}=\sqrt{(l\pm m+1)(l\mp m)}$. Also, we consider the Mie scattering coefficients for a self-standing homogenous sphere of radius a and permittivity ϵ_d centered at the origin, which are given by

$$t_l^M = \frac{-j_l(\rho_0)\rho_1 j_l'(\rho_1)+\rho_0 j_l'(\rho_0)j_l(\rho_1)}{h_l^{(+)}(\rho_0)\rho_1 j_l'(\rho_1)-\rho_0[h_l^{(+)}(\rho_0)]'j_l(\rho_1)}, \tag{S5}$$

$$t_l^E = \frac{-j_l(\rho_0)\tilde{j}_l(\rho_1)'+\epsilon_d\tilde{j}_l(\rho_0)'j_l(\rho_1)}{h_l^{(+)}(\rho_0)\tilde{j}_l(\rho_1)'-\epsilon_d\tilde{h}_l^{(+)}(\rho_0)'j_l(\rho_1)}, \tag{S6}$$

where $\rho_0=\omega a/c$, $\rho_1=\sqrt{\epsilon_d}\omega a/c$, j_l and $h_l^{(+)}$ are spherical Bessel and Hankel functions [5], we have defined the functions $\tilde{j}_l(\rho)=\rho\,j_l(\rho)$ and $\tilde{h}_l^{(+)}(\rho)=\rho\,h_l^{(+)}(\rho)$, and the prime denotes differentiation

with respect to the argument. Finally, the incident field supplied by the electron is represented through the coefficients [6]

$$\begin{bmatrix} \psi_{lm}^{M,\mathrm{ext}} \\ \\ \psi_{lm}^{E,\mathrm{ext}} \end{bmatrix} = \frac{-2\pi\, e\, \mathrm{i}^{1-l}\omega}{l(l+1)\, c^2}\, K_m\left[\frac{\omega R_e}{v\gamma}\right] \mathrm{e}^{-\mathrm{i}m\varphi_e} \times \begin{bmatrix} 2mA_{lm}v/c \\ \\ B_{lm}/\gamma \end{bmatrix}, \tag{S7}$$

where (R_e, φ_e) are the polar coordinates of the electron transverse vector (x_e, y_e), K_m is the modified Bessel function of order m, $\gamma = 1/\sqrt{1-(v/c)^2}$ is the Lorentz factor, and the coefficients A_{lm} and B_{lm} only depend on the normalized electron velocity v/c. More precisely [7],

$$A_{lm} = \mathrm{i}^{l+m}(2m-1)!!\sqrt{\frac{(2l+1)}{\pi}\frac{(l-m)!}{(l+m)!}}\,\frac{(c/v)^{m+1}}{\gamma^m}\, C_{l-m}^{m+1/2}(c/v), \qquad m \geq 0, \tag{S8}$$

$$B_{lm} = A_{l,m+1}\sqrt{(l+m+1)(l-m)} \;-\; A_{l,m-1}\sqrt{(l-m+1)(l+m)}, \tag{S9}$$

and C_ν^μ are Gegenbauer polynomials [8]. We use the property $A_{l,-m} = (-1)^m A_{lm}$ to calculate the A_{lm} coefficients for negative m.

These results allow us to calculate the EEGS probability $|\beta_{\mathrm{EEGS}}(\omega)|^2$ for an electron moving downwards and passing close to a homogeneous sphere under plane-wave light irradiation from Eq. (S2) by using the CL amplitude $\mathbf{f}_{\hat{\mathbf{r}}}^{\mathrm{CL}}(\omega)$ given by Eq. (S3) for an electron moving along the positive z axis (see Fig. S3).

B. Illumination by means of a parabolic mirror

Illumination of the specimen in the microscope is introduced through a parabolic aluminum mirror, producing a focal spot that can be regarded as a superposition of plane waves resulting from reflection of the incident laser on different regions of the metal surface. We model the system by describing the reflection from each mirror surface position $\mathbf{s}$ through the Fresnel coefficients of a tangent planar mirror at that position.

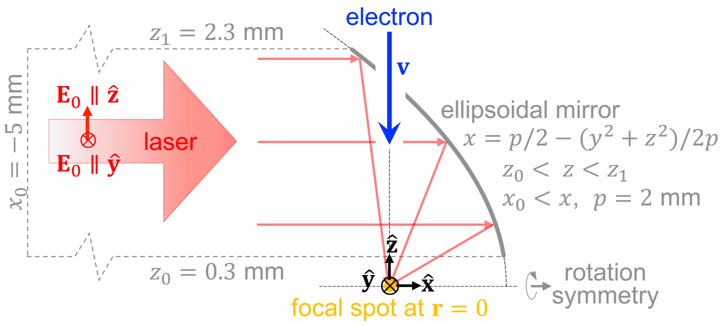

FIG. S4: **Illumination configuration used in the present work.** We assume light incident from the left and propagating towards the positive x direction, the e-beam is injected through a hole and moves downwards along the negative z direction, and the specimen is placed near the focal spot at $\mathbf{r} = 0$. The mirror surface is defined by the equation $x = p/2 - (y^2 + z^2)/2p$. In the present experiment, we have $p = 2$ mm, and the mirror surface is limited by the conditions $x > x_0 = -5$ mm and $z_0 = 0.3$ mm $< z < z_1 = 2.3$ mm. The hole is ignored in our calculations. In this work, we consider a laser field amplitude $\mathbf{E}_0$ oriented along either the y or z directions.

In what follows, we consider the e-beam to move along the negative z direction and the parabolic mirror to be illuminated by a laser plane wave propagating along the positive x direction, as illustrated in Fig. S4. To obtain the field reaching the specimen region after reflection by the mirror, we assimilate the response of each surface element to effective $\mathbf{s}$-dependent electric and magnetic dipole densities $\mathbf{p_s}$ and $\mathbf{m_s}$, the magnitudes of which are obtained in the limit of a planar mirror, as we mentioned above.

We now analyze the equivalent dipole sources produced by reflection on a planar mirror. More precisely, for an incident light plane wave $\mathbf{E}^i \mathrm{e}^{\mathrm{i}\mathbf{k}^i\cdot\mathbf{r}}$ of wave vector $\mathbf{k}^i$ and electric field amplitude $\mathbf{E}^i$, the field reflected by a planar mirror that contains the origin $\mathbf{r}=0$ and has an outer normal unit vector $\hat{\mathbf{n}}$ is given by $\mathbf{E}^r \mathrm{e}^{\mathrm{i}\mathbf{k}^r\cdot\mathbf{r}}$, where $\mathbf{k}^r=\mathbf{k}^i-2(\mathbf{k}^i\cdot\hat{\mathbf{n}})\hat{\mathbf{n}}$ is the reflected wave vector, and $\mathbf{E}^r = r_{\rm s}(\mathbf{E}^i\cdot\hat{\mathbf{e}}_s)\,\hat{\mathbf{e}}_s + r_{\rm p}(\mathbf{E}^i\cdot\hat{\mathbf{e}}_p^i)\,\hat{\mathbf{e}}_p^r$ is the reflected field amplitude written in terms of the Fresnel coefficients $r_{\rm s}$ and $r_{\rm p}$ for s and p polarization. Here, $\hat{\mathbf{e}}_s$ and $\hat{\mathbf{e}}_p^\nu$ are the corresponding unit polarization vectors. We note that the p-polarization vector $\hat{\mathbf{e}}_p^\nu$ is different for incidence ($\nu=i$) and reflection ($\nu=r$) directions (see below). Translational invariance of the planar mirror allows us to incorporate the $\mathbf{s}$ dependence of the effective dipoles through $\mathbf{p_s}=\mathbf{p}\,\mathrm{e}^{\mathrm{i}\mathbf{k}_\parallel\cdot\mathbf{s}}$ and $\mathbf{m_s}=\mathbf{m}\,\mathrm{e}^{\mathrm{i}\mathbf{k}_\parallel\cdot\mathbf{s}}$, where $\mathbf{k}_\parallel=\mathbf{k}^i-(\mathbf{k}^i\cdot\hat{\mathbf{n}})\hat{\mathbf{n}}$ is the in-plane component of the incident wave vector. In terms of these effective sources, and assuming a permittivity ϵ_0 ($=1$ in the present work) in the medium outside the mirror, the reflected field becomes

$$\int d^2\mathbf{s}\,\left[k^2\,\mathbf{p_s}+\frac{1}{\epsilon_0}(\mathbf{p_s}\cdot\nabla_\mathbf{r})\nabla_\mathbf{r}-\mathrm{i}k\,\mathbf{m_s}\times\nabla_\mathbf{r}\right]\frac{\mathrm{e}^{\mathrm{i}k\sqrt{\epsilon_0}|\mathbf{r}-\mathbf{s}|}}{|\mathbf{r}-\mathbf{s}|}=\frac{2\pi\mathrm{i}k^2}{k_\perp}\,\mathrm{e}^{\mathrm{i}\mathbf{k}^r\cdot\mathbf{r}}\left[\mathbf{p}-(\mathbf{p}\cdot\hat{\mathbf{k}}^r)\hat{\mathbf{k}}^r+\sqrt{\epsilon_0}\,\mathbf{m}\times\hat{\mathbf{k}}^r\right], \tag{S10}$$

where $k=\omega/c$, $k_\perp=-\mathbf{k}^i\cdot\hat{\mathbf{n}}$, and the $\mathbf{s}$ integral is extended over the plane normal to $\hat{\mathbf{n}}$. Comparing this result with the reflected field $\mathbf{E}^r\mathrm{e}^{\mathrm{i}\mathbf{k}^r\cdot\mathbf{r}}$ and noticing that $\mathbf{E}^r\cdot\mathbf{k}^r=0$, we find that we can take $\mathbf{m}=0$ and $\mathbf{p}=(-\mathrm{i}k_\perp/2\pi k^2)\,\mathbf{E}^r$. Finally, inserting the expression given above for $\mathbf{E}^r$ in terms of the Fresnel coefficients, we have $\mathbf{p}=(-\mathrm{i}k_\perp/2\pi k^2)\left[r_{\rm s}(\mathbf{E}^i\cdot\hat{\mathbf{e}}_s)\,\hat{\mathbf{e}}_s+r_{\rm p}(\mathbf{E}^i\cdot\hat{\mathbf{e}}_p^i)\,\hat{\mathbf{e}}_p^r\right]$.

For a parabolic mirror defined by $x=p/2-(y^2+z^2)/2p$ (focal distance $p/2$), as shown in Fig. S4, we define the coordinates $\mathbf{r}_\perp=(y,z)$ in the transverse plane relative to the rotation axis x and write the elements involved in the calculation of the reflected field at each position $\mathbf{s}$ as

$$\hat{\mathbf{n}}=-(\mathbf{r}_\perp+p\hat{\mathbf{x}})/\sqrt{r_\perp^2+p^2}, \tag{S11a}$$

$$\hat{\mathbf{k}}^i=\hat{\mathbf{x}}, \tag{S11b}$$

$$\hat{\mathbf{k}}^r=[(r_\perp^2-p^2)\hat{\mathbf{x}}-2p\mathbf{r}_\perp)]/(r_\perp^2+p^2), \tag{S11c}$$

$$\hat{\mathbf{e}}_s=(-z\hat{\mathbf{y}}+y\hat{\mathbf{z}})/r_\perp, \tag{S11d}$$

$$\hat{\mathbf{e}}_p^i=\hat{\mathbf{r}}_\perp, \tag{S11e}$$

$$\hat{\mathbf{e}}_p^r=[(r_\perp^2-p^2)\mathbf{r}_\perp+2pr_\perp\hat{\mathbf{x}})]/(r_\perp^2+p^2), \tag{S11f}$$

and $\mathbf{k}^\nu=k\sqrt{\epsilon_0}\,\hat{\mathbf{k}}^\nu$. In addition, the normal wave vector becomes $k_\perp=k\sqrt{\epsilon_0}\,p/\sqrt{r_\perp^2+p^2}$. Using these elements, we can calculate the electric field $\mathbf{E}(\mathbf{r})$ produced by the mirror at a position $\mathbf{r}$ near the focal point ($\mathbf{r}=0$) from an expression similar to the left-hand side of Eq. (S10), but now with

$\mathbf{s}$ integrated over the surface of the curved mirror. More precisely,

$$\mathbf{E}(\mathbf{r}_f) = \int_{\rm mirror} d^2\mathbf{s} \left[k^2\,\mathbf{p}_\mathbf{s} + \frac{1}{\epsilon_0}(\mathbf{p}_\mathbf{s}\cdot\nabla_{\mathbf{r}_f})\nabla_{\mathbf{r}_f}\right] \frac{{\rm e}^{{\rm i}k\sqrt{\epsilon_0}|\mathbf{r}_f-\mathbf{s}|}}{|\mathbf{r}_f-\mathbf{s}|} \tag{S12a}$$

$$\approx {\rm e}^{{\rm i}kp\sqrt{\epsilon_0}} \int_{\rm mirror} d^2\mathbf{s} \left[k^2\,\mathbf{p}_\mathbf{s} - \frac{1}{\epsilon_0}(\mathbf{p}_\mathbf{s}\cdot\mathbf{k}^r)\mathbf{k}^r\right] \frac{{\rm e}^{{\rm i}\mathbf{k}^r\cdot\mathbf{r}_f}}{s} \tag{S12b}$$

$$= -{\rm e}^{{\rm i}kp\sqrt{\epsilon_0}}\,\frac{\rm i}{2\pi}\int_{\rm mirror} d^2\mathbf{s}\,\frac{k_\perp}{s}\,{\rm e}^{{\rm i}\mathbf{k}^r\cdot\mathbf{r}_f}\left[r_{\rm s}(\mathbf{E}_0\cdot\hat{\mathbf{e}}_s)\,\hat{\mathbf{e}}_s + r_{\rm p}(\mathbf{E}_0\cdot\hat{\mathbf{e}}_p^i)\,\hat{\mathbf{e}}_p^r\right] \tag{S12c}$$

$$= -{\rm e}^{{\rm i}kp\sqrt{\epsilon_0}}\,\frac{{\rm i}k\sqrt{\epsilon_0}}{2\pi}\int_{\rm mirror} d\mathbf{r}_\perp\,\frac{1}{s}\,{\rm e}^{{\rm i}\mathbf{k}^r\cdot\mathbf{r}_f}\left[r_{\rm s}(\mathbf{E}_0\cdot\hat{\mathbf{e}}_s)\,\hat{\mathbf{e}}_s + r_{\rm p}(\mathbf{E}_0\cdot\hat{\mathbf{e}}_p^i)\,\hat{\mathbf{e}}_p^r\right], \tag{S12d}$$

where (S12b) is obtained from (S12a) by adopting the $kp \gg 1$ limit (i.e., considering that the focal distance $p/2$ is large compared with the light wavelength) and using the relation $\hat{\mathbf{k}}^r = -\hat{\mathbf{s}}$ to connect the reflected wave vector to the direction of the surface position $\mathbf{s}$ (as seen from the focus) in the integral. In doing so, we are assuming an incident plane wave $\mathbf{E}_0\,{\rm e}^{ikx}$ illuminating the mirror, such that the sum of light propagation distances from the $x=0$ plane to the mirror surface and from this to the focal point amounts to a path length given by p and emerging as an irrelevant global phase factor in Eq. (S12b). Then, Eq. (S12c) is obtained by noticing that $\mathbf{p}_\mathbf{s}\cdot\mathbf{k}^r = 0$ because $\mathbf{p}_\mathbf{s} \propto \mathbf{E}^r$ and the reflected field must be transverse. In addition, we insert the explicit expression for $\mathbf{p}$ derived above for the planar mirror in terms of the Fresnel coefficients, with $\mathbf{E}^i$ substituted by $\mathbf{E}_0$. We note that the $\mathbf{s}$-dependent phase in $\mathbf{E}^i$ is already included in the global phase factor ${\rm e}^{ikp\sqrt{\epsilon_0}}$. Finally, Eq. (S12d) results from a change of integration variables $\mathbf{s}\to\mathbf{r}_\perp$ after noticing that the corresponding Jacobian times $k_\perp$ reduces to $k\sqrt{\epsilon_0}$. The electric field in the focal region is then given by Eq. (S12d) using the quantities defined in Eqs. (S11) in terms of $\mathbf{r}_\perp = (y,z)$ as well as $s = (p^2 + r_\perp^2)/2p$. Also, we use the Fresnel coefficients

$$r_{\rm s} = (k_\perp - k'_\perp)/(k_\perp + k'_\perp), \tag{S13a}$$

$$r_{\rm p} = (\epsilon_m k_\perp - \epsilon_0 k'_\perp)/(\epsilon_m k_\perp + \epsilon_0 k'_\perp), \tag{S13b}$$

where $k'_\perp = \sqrt{k^2(\epsilon_m-\epsilon_0)+k_\perp^2}$ and ϵ_m is the metal permittivity.

In our experimental setup, we have an aluminum mirror ($\epsilon_m(\omega)$ taken from optical data [9]) in vacuum ($\epsilon_0 = 1$) with a focal distance of 1 mm (i.e., $p = 2$ mm) and the metal surface limited by the conditions stated in Fig. S4. We adopt these parameters throughout the present paper. Then, the focal spot size is about 1 μm, with the actual shape depending on the polarization of the incident light, as shown in Fig. S5.

C. EEGS under illumination by a parabolic mirror

Like the focal spot, the electron–light coupling coefficient $\beta_{\rm EEGS}(\omega)$ can also be regarded as a superposition of contributions due to different reflected plane waves. Using Eq. (S2) for the contribution of each of these waves, integrating over the mirror surface just like in Eq. (S12d), and ignoring any global phase, we find

$$\beta_{\rm EEGS}(\omega) = \frac{c}{\pi\hbar\omega}\int_{\rm mirror} d\mathbf{r}_\perp\,\frac{p}{p^2+r_\perp^2}\,\mathbf{f}^{\rm CL}_{\hat{\mathbf{s}}}(\omega)\cdot\left[r_{\rm s}(\mathbf{E}_0\cdot\hat{\mathbf{e}}_s)\,\hat{\mathbf{e}}_s + r_{\rm p}(\mathbf{E}_0\cdot\hat{\mathbf{e}}_p^i)\,\hat{\mathbf{e}}_p^r\right] \tag{S14a}$$

$$= \frac{c}{\pi\hbar\omega}\int_{z_0}^{z_1} dz \int_{-y_0(z)}^{y_0(z)} dy\,\frac{p}{p^2+r_\perp^2}\,\mathbf{f}^{\rm CL}_{\hat{\mathbf{s}}}(\omega)\cdot\left[r_{\rm s}(\mathbf{E}_0\cdot\hat{\mathbf{e}}_s)\,\hat{\mathbf{e}}_s + r_{\rm p}(\mathbf{E}_0\cdot\hat{\mathbf{e}}_p^i)\,\hat{\mathbf{e}}_p^r\right], \tag{S14b}$$

involving $\hat{\mathbf{s}} = -\hat{\mathbf{k}}^r$ as well as different quantities defined in Eqs. (S11) and (S13). We remark that Eq. (S14a) is general for any parabolic mirror geometry, while Eq. (S14b) is specialized to our

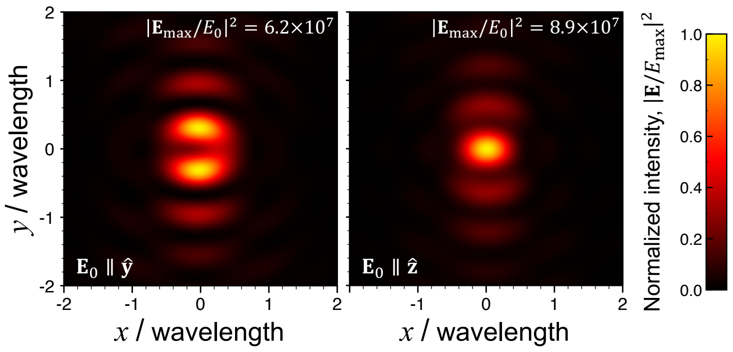

FIG. S5: **Focal spot produced in the $z = 0$ plane by the mirror in Fig. S4.** We consider incident light polarization along either the y (left) or the z (right) directions. The electric field intensity is normalized to the maximum $|E_{\rm max}|^2$, with values of the latter relative to the incident field intensity $|E_0|^2$ also indicated. The calculation is made for an aluminum mirror at a photon energy of 2 eV (i.e., a wavelength $\lambda = 620$ nm). Similar results are obtained when assimilating the metal to a perfect conductor, so these results describe well the focal spot at frequencies up to the visible range. The mirror has a projected area on the yz plane of 18.74 mm^2 = $4.87 \times 10^7\,\lambda^2$. The effective projected area including absorption in aluminum is 16.51 mm^2 and 17.35 mm^2 for $\mathbf{E}_0 \parallel \hat{\mathbf{y}}$ and $\mathbf{E}_0 \parallel \hat{\mathbf{z}}$, respectively.

mirror dimensions, as described in Fig. S4, such that the limits of integration are $z_0 = 0.3$ mm, $z_1 = 2.3$ mm, and $y_0(z) = \sqrt{p^2 - 2px_0 - z^2}$ with $x_0 = -5$ mm and $p = 2$ mm. Also, Eqs. (S14) can be applied to any specimen by plugging the corresponding CL amplitude $\mathbf{f}^{\rm CL}_{\hat{\mathbf{s}}}(\omega)$. In the present study, we calculate the latter for a sphere by means of Eq. (S3).

S3. MIE MODES IN LARGE DIELECTRIC SPHERES

To characterize the large dielectric spheres studied in this work, we first calculate their optical extinction cross sections as a function of light wavelength $\lambda = 2\pi c/\omega$ from Mie theory [10] using the expression

$$\sigma^{\rm ext}(\lambda) = \frac{\lambda}{2\pi\sqrt{\epsilon_0}} \sum_{l=1}^{\infty} (2l+1)\big[{\rm Im}\{t_l^M\} + {\rm Im}\{t_l^E\}\big], \tag{S15}$$

where we set $\epsilon_0 = 1$ (particle in vacuum).

For the 4122 nm silica sphere, we fine-tune the diameter to match the two prominent resonances observed in the EEGS/EELS/CL experiments. The results presented in Fig. S6 show that such resonances have electric polarization with multipolar orders $l = 27$ and 26 at wavelengths of $\sim$ 582 nm and $\sim$ 602 nm, respectively. There is an additional magnetic resonance at $\sim$ 593 nm with $l = 27$, which is missed in the experiments for symmetry reasons, as we argue below. The quality factors of these modes are ~ 1500, substantially larger than those observed in experiment ~ 200, in which losses at the support or due to surface contamination can affect the actual resonances.

A larger polysterene (PS) sphere is also studied, for which we predict modes with quality factors reaching 10^6 when the particle is self-standing (Fig. S7d). This is in contrast to the values $\sim 10^4$ observed in experiment, a disagreement that could be attributed to losses originating in the effect of the supporting film. In addition, we cannot find a good match between the calculated Mie resonances and the three peaks reported in Fig. 4 of the main text even when fine-tuning the sphere diameter. A possible reason for this discrepancy is that the particle (including the effect of the support) deviates from the spherical geometry assumed in Mie theory, such that the three observed

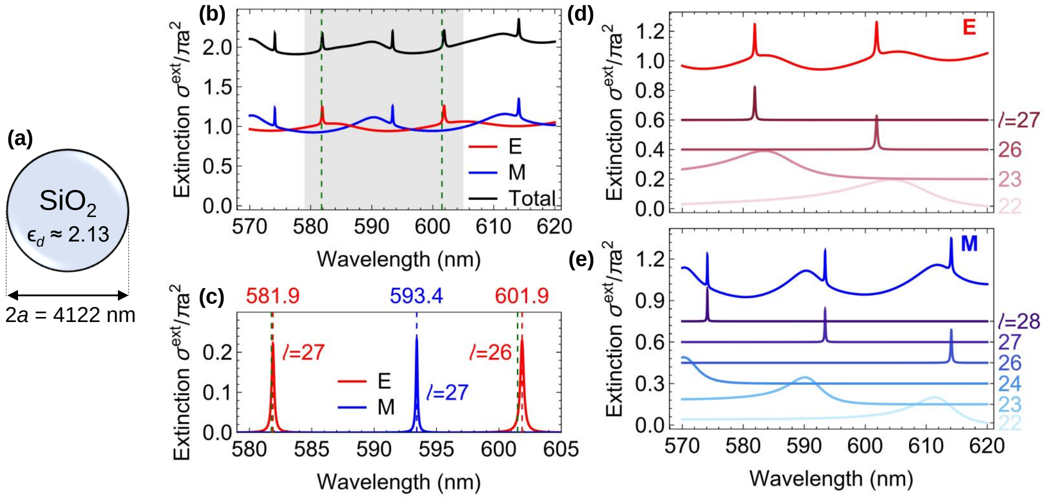

FIG. S6: **Extinction cross-section spectrum of a large SiO_2 sphere.** **(a)** Sketch of the sphere with a diameter $2a = 4122$ nm and a permittivity $\epsilon_d \approx 2.13$. **(b)** Total optical extinction cross section $\sigma^{\rm ext}$ (black curve) normalized to the geometrical cross section πa^2, along with the corresponding partial contributions due to electric (red) and magnetic (blue) modes, as a function of light wavelength. The vertical dashed lines indicate the two resonance wavelengths observed in the experimental results of Fig. 2 in the main text. **(c)** Partial extinction cross sections within the shaded region of panel (b), allowing us to identify two electric modes with $l = 26$ and 27 (red), as well as a magnetic mode with $l = 27$ (blue). The vertical dashed green lines are the same as in panel (b). **(d)** Total extinction cross section due to electric modes (top curve) and partial contributions from different multipolar orders l (see labels on the right axis). A vertical offset between different curves is introduced for clarity. **(e)** Same as panel (d), but for magnetic modes.

peaks originate in the splitting of a single high-order multipolar mode relative of the idealized sphere. We therefore search for a possible Mie resonance in the sphere that has large optical strength in the measured spectral range. To compare with experiment, we introduce a factor $1 + 10^{-4}\,{\rm i}$ in the material permittivity, so that the maximum quality factors of the Mie resonances are brought down to $\sim 10^4$ (see Fig. S7d). The resulting multipolar decomposition of this slightly lossy sphere reveals a magnetic resonance of multipolar order $l = 51$ and an electric one of order $l = 50$ near the observed features (Fig. S7c), so these are possible candidates to explain the measurements if a small shift and splitting is produced by the lack of sphericity or the effect of the support. Such effects would be less relevant in the smaller silica sphere studied above because of the larger width of its resonances.

S4. EEGS IN A LARGE SILICA SPHERE

We start by calculating the EELS probability for the 4122 nm silica sphere considered in Sec. S3 (Fig. S8a), which coincides with the CL emission probability because the material is lossless. Using the elements introduced in Sec. S2 A, these probabilities are given by [2]

$$\begin{bmatrix} \Gamma_{\rm EELS}(\omega) \\ \\ \Gamma_{\rm CL}(\omega) \end{bmatrix} = \frac{\alpha}{\omega}\sum_{l=1}^{\infty}\sum_{m=-l}^{l}\frac{1}{l(l+1)}K_m^2\left(\frac{\omega R_e}{v\gamma}\right)\left(\frac{4m^2v^2}{c^2}|A_{lm}|^2\begin{bmatrix} {\rm Im}\{t_l^M\} \\ \\ |t_l^M|^2 \end{bmatrix} + \frac{1}{\gamma^2}|B_{lm}|^2\begin{bmatrix} {\rm Im}\{t_l^E\} \\ \\ |t_l^E|^2 \end{bmatrix}\right), \tag{S16}$$

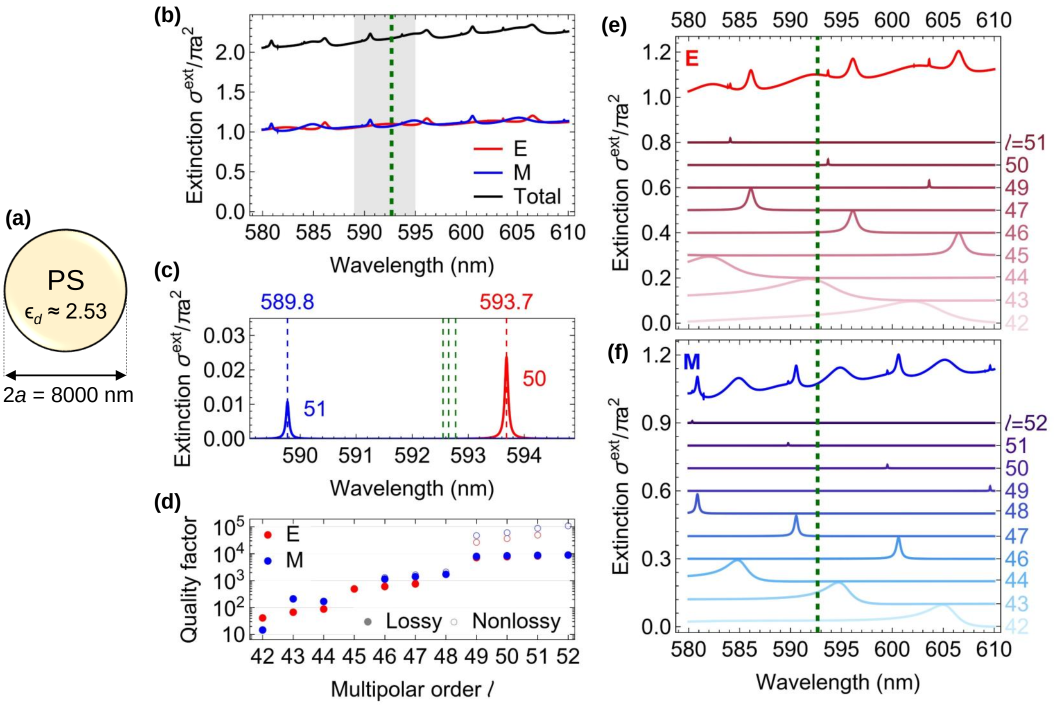

FIG. S7: **Extinction cross-section spectrum of a large polystyrene (PS) sphere.** **(a)** Sketch of the sphere with a diameter $2a = 8000\,$nm and a permittivity $\epsilon_d \approx 2.53 \times (1 + i\eta)$, where losses are phenomenologically introduced through a small imaginary part $\eta = 10^{-4}$ in the multiplying factor. **(b)** Total optical extinction cross section $\sigma^{\rm ext}$ (black curve) normalized to the geometrical cross section πa^2, along with the corresponding partial contributions due to electric (red) and magnetic (blue) modes, as a function of light wavelength. The vertical dashed lines indicate the position of the resonances observed in the experimental measurements (see Fig. 3 in the main text). **(c)** Partial cross sections contributed by magnetic modes with $l = 46, 47$ (blue) and electric modes with $l =$ 45-47 (red), giving rise to the resonances observed in panel (b). The vertical dashed green lines are the same as in panel (b). **(d)** Quality factors of electric (red) and magnetic (blue) Mie resonances as a function of their multipolar order l. Solid and open circles are calculated with $\eta = 10^{-4}$ (partially lossy PS) and $\eta = 0$ (nonlossy PS), respectively. **(e)** Total extinction cross section due to electric modes (top curve) and partial contributions from different multipolar orders l (see labels on the right axis). A vertical offset between different curves is introduced for clarity. **(f)** Same as panel (e), but for magnetic modes.

such that the total frequency-integrated probability per electron is $\int_0^\infty d\omega\, \Gamma_{\rm EELS,CL}(\omega)$. Incidentally, these expressions are made unit-independent by using the fine structure constant $\alpha \approx 1/137$. The calculated spectrum is dominated by the two electric Mie modes discussed in Fig. S6c, with an additional small contribution from the intermediate magnetic mode.

Likewise, the EEGS probability $|\beta_{\rm EGGS}(\omega)|^2$, obtained from Eqs. (S3) and (S14b), is dominated by the same two electric modes (Fig. S8b). In particular, for the configuration A (see inset), the magnetic mode only produces a residual feature in the EEGS spectral profile. Incidentally, the effect of light concentration by the mirror gives rise to an enhancement by five orders of magnitude in the EEGS probability relative to direct light plane-wave illumination for a given value of the light intensity I_0 (cf. Fig. S8b and Fig. S8c).

In Fig. S9, we present spatial maps of the calculated EEGS probability $|\beta_{\rm EGGS}(\omega)|^2$ for the 4122 nm silica sphere. Each pixel in the map corresponds to a different e-beam position for a fixed

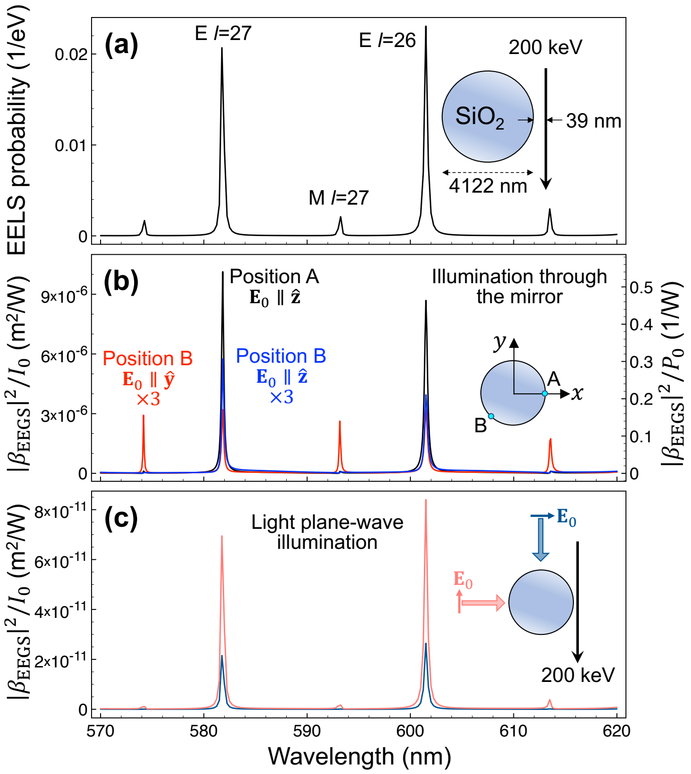

FIG. S8: **EELS and EEGS in the silica sphere of Fig. S6**. **(a)** EELS probability calculated from Eq. (S16) as a function of light wavelength $2\pi c/\omega$. **(b)** EEGS probability $|\beta_{\rm EGGS}(\omega)|^2$ obtained from Eq. (S14b) and normalized either to the laser intensity I_0 that is incident on the mirror described in Fig. S4 ($|\beta_{\rm EGGS}(\omega)|^2/I_0$, left vertical scale) or to the laser power P_0 ($|\beta_{\rm EGGS}(\omega)|^2/P_0$, right vertical scale, with $P_0 = I_0 A$, where $A = 18.74\,{\rm mm}^2$ is the area of the mirror projection on the yz plane), with the laser focal spot aimed at positions that are 57 nm inside the projected sphere contour; the EEGS probability is calculated for two different azimuthal locations of the focal spot and the e-beam (A and B in the inset, with the focal spot and the e-beam sharing the same azimuthal position in each case), it vanishes in A for incident light polarized along y due to symmetry considerations, and it is multiplied by a factor of 3 in B. The orientation of the Cartesian axes is the same as in Fig. S4. **(c)** EEGS probability computed from Eq. (S2) and normalized to the incident light intensity for plane-wave illumination with two different directions of light incidence (see color-coordinated thick arrows in the inset). Electrons have an energy of 200 keV and pass at a distance of 39 nm from the sphere surface in all cases.

light focal spot (blue dots). We only consider e-beam positions that do not intersect the sphere. Results are presented for the three Mie modes discussed in Fig. S6c and for four different positions of the focal spot. These plots demonstrate that substantial electron-light coupling takes place even when the e-beam passes at a large distant (but still near the sphere surface) relative to the focal spot. This is a manifestation of the delocalized nature of the Mie modes under consideration.

Incidentally, when reducing the size of the mirror, the EEGS probability $|\beta_{\rm EGGS}(\omega)|^2$ decreases roughly in proportion to the mirror area, as shown in Fig. S10. However, when normalized to the total laser power P_0 collected by the mirror (see upper color scale), we find a similar order of magnitude of $|\beta_{\rm EGGS}(\omega)|^2/P_0$ as for the larger mirror, indicating that the electron couples to Mie modes that are relatively delocalized, and thus, although a larger mirror is beneficial for delivering more power in practice, the size of the focal spot is not too relevant for the present specimen.

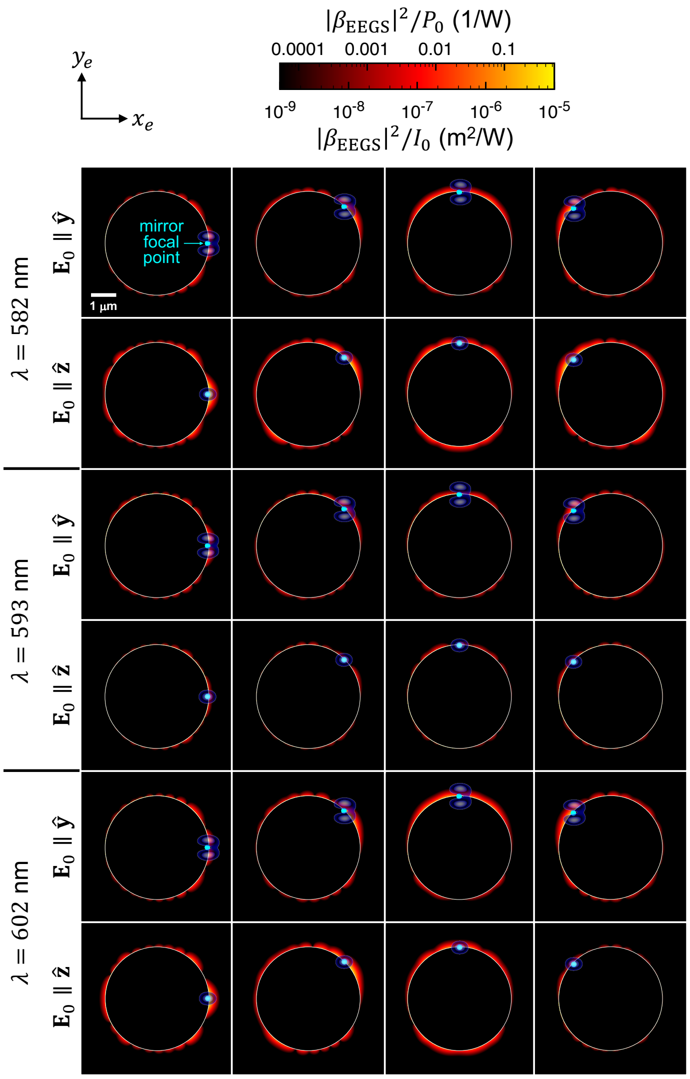

FIG. S9: **Spatial maps of the EEGS probability for the silica sphere of Fig. S6 (4122 nm diameter).** We plot $|\beta_{\rm EGGS}(\omega)|^2$ as a function of e-beam position (x_e, y_e) (see upper-left frame, coordinated with Fig. S4) for illumination on resonance with the three Mie modes of Fig. S6c (see the corresponding wavelengths $\lambda = 2\pi c/\omega$ on the left) and 200 keV electron energy. We consider two different orientations of the incident field $\mathbf{E}_0$ referred to the mirror in Fig. S4 ($\parallel \hat{\mathbf{y}}$ or $\parallel \hat{\mathbf{z}}$, see labels on the left). Results are presented for four different positions of the laser focal spot (blue dots) relative to the sphere (white contours). The EEGS probability $|\beta_{\rm EGGS}(\omega)|^2$ (color log scale) is normalized to the laser intensity $I_0 = c|E_0|^2/2\pi$ incident on the mirror (i.e., $|\beta_{\rm EGGS}(\omega)|^2/I_0$, lower scale) and the laser power $P_0 = I_0 A$ incident on the mirror (i.e., $|\beta_{\rm EGGS}(\omega)|^2/P_0$, upper scale), where $A = 18.74\,{\rm mm}^2$ is the area of the mirror projection on the yz plane. We only plot $|\beta_{\rm EGGS}(\omega)|^2$ for e-beam positions that do not intersect the particle. The focal-spot intensity at $z = 0$ (taken from Fig. S5) is superimposed for each $\mathbf{E}_0$ orientation (color scale from maximum intensity (blue) down to a fraction $1/e$ of it (white contour)).

Another signature of optical field delocalization is found by examining the EEGS spectra produced at a constant e-beam position as we move the optical spot. The results shown in Fig. S11 reveal

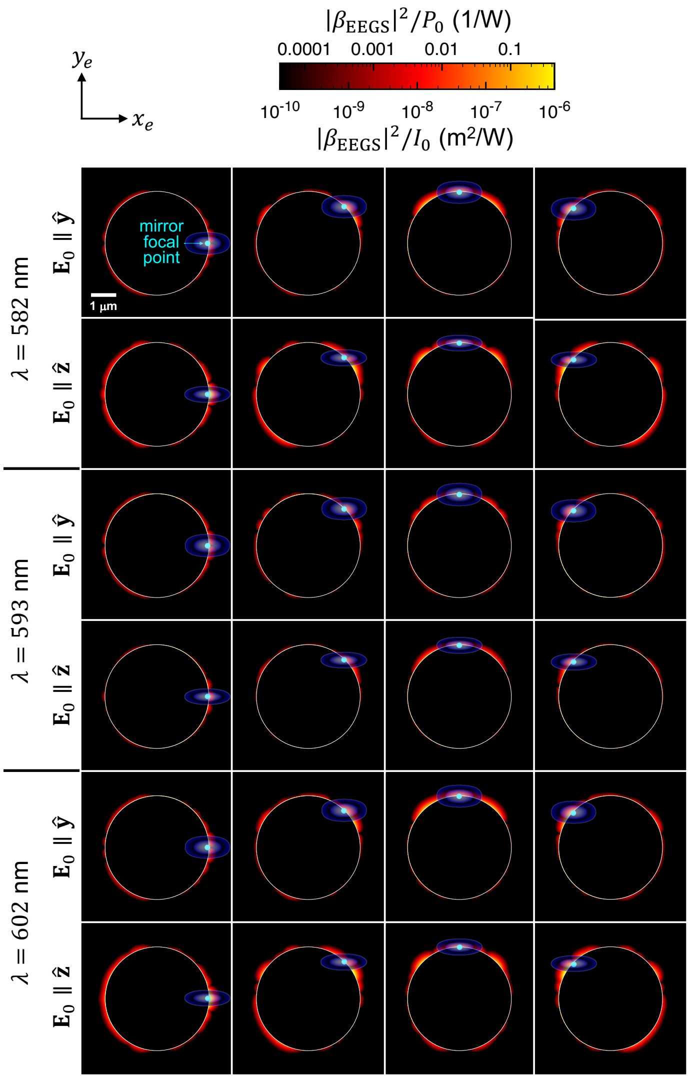

FIG. S10: **Same as Fig. S9, but with a smaller mirror defined by $x_0 = 0.5\,$mm (see Fig. S4).** The smaller mirror area ($A = 2.30\,\mathrm{mm}^2$ when projected on the yz plane) produces larger laser focal spots. The color scale for $|\beta_{\rm EGGS}(\omega)|^2/P_0$ runs over similar values as in Fig. S9 (i.e., we have a similar coupling to the electron for the same power incident on the mirror in each case), while the scale for $|\beta_{\rm EGGS}(\omega)|^2/P_0$ shows numbers that are roughly one order of magnitude smaller than in Fig. S9, in agreement with the ratio of projected mirror areas between both mirrors.

two interesting trends. (1) When the e-beam and laser spot cross the same azimuthal direction (the positive x axis in Fig. S11a), the EEGS probability takes comparatively large values, which tend to increase as the optical spot is placed further apart from the sphere center, such that it is able to excite Mie resonances of higher angular momentum following the rule $l \sim \omega R_f/c$, which yields an orbital number $l \sim 26$ at $R_l = 2.5\,\mu$m, matching well the two prominent modes at $l = 26$ and 27 observed in Fig. S11 (see Fig. S8). (2) In addition, when the azimuthal positions of the e-beam and optical spots are different (Fig. S11b,c), such radial dependence becomes much weaker and the

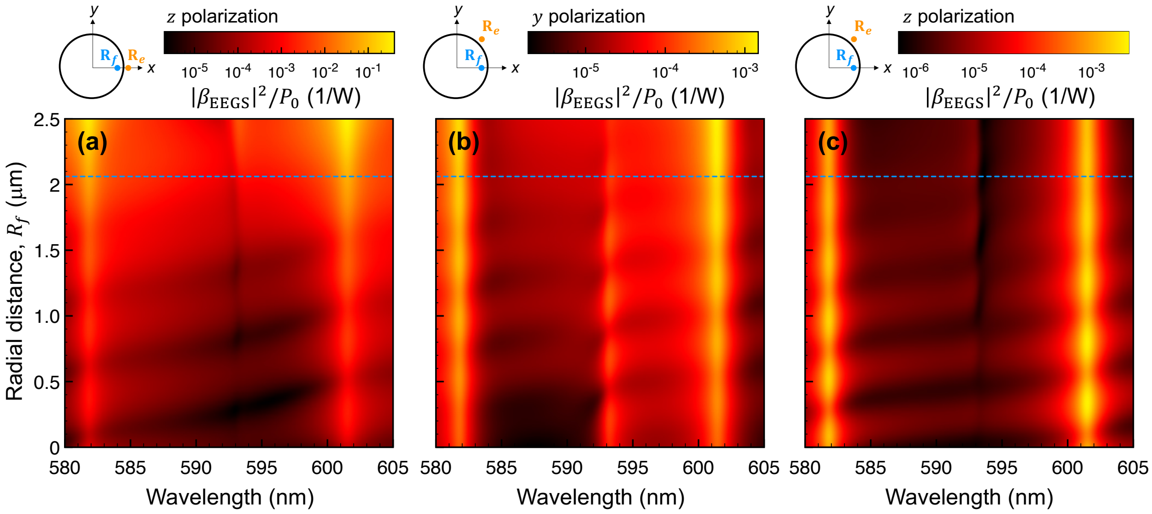

FIG. S11: **Dependence of the EEGS spectra on the position of the optical focal spot.** We plot the EEGS probability for the silica sphere of Fig. S6 (4122 nm diameter) as a function of laser wavelength (horizontal axes) and radial position of the laser focal spot R_f. The latter is placed along the positive x axis. The sphere edge is indicated by dashed horizontal lines. The e-beam passes 50 nm outside the sphere surface, crossing either the x axis in (a) or a line that forms 45° with the x axis in (b,c) (see upper-left insets). The laser is polarized along z in (a,c) and along y in (b) (see Fig. S4). The silica permittivity is supplemented with an additional imaginary part of 10^{-3} i to slightly broaden the resonances.

mode wavelength	4122 nm silica sphere grazing	4122 nm silica sphere 50 nm distance	8636 nm PS sphere grazing	8636 nm PS sphere 50 nm distance
582 nm	0.0100	0.0031	-	-
593 nm	0.0032	0.0096	-	-
602 nm	0.0219	0.0070	-	-
592 nm	-	-	8.5×10^{-5}	2.8×10^{-5}
594 nm	-	-	0.0003	0.0001

TABLE S1: CL probabilities for 200 keV electrons passing either grazingly or at a distance of 50 nm from the surface of the 4122 nm sphere of Fig. S6 or the 8636 nm PS sphere of Fig. S7. The probabilities are integrated over the spectral peaks at the wavelengths indicated in the left column. Incidentally, the CL probability for the PS sphere is nearly independent on the parameter η used to introduce losses (see Fig. S7) when comparing $\eta = 0$ and $\eta = 10^{-4}$, although the EELS probability is substantially increased with the latter choice.

EEGS probability is comparatively reduced, indicating that the e-beam misses the path followed by ballistic-like whispering gallery modes launched along a sphere meridian when the laser beam is grazingly incident.

Finally, we compare the CL emission probability integrated over frequency within the Mie resonances under consideration for the silica and PS spheres considered in Figs. S6 and S7. The results,

presented in Table S1, show that the probability is much higher in the silica particle, thus providing an additional piece of information to explain why only this one can be resolved in our CL experiments.

S5. ON THE QUESTION OF SPECTRAL RESOLUTION AND SIGNAL STRENGTH IN EELS, CL, AND EEGS

The spectral resolution in EELS is generally limited by the width of the zero-loss peak and the energy analyzer. In contrast, the resolution in CL depends on the precision of the light spectrometer. Finally, the EEGS resolution is controlled by the width of the external laser. One could argue that CL and EEGS should be ultimately comparable in precision, as they rely on optical tools. However, a problem arises regarding the ability of these techniques to detect narrow resonances: indeed, our experimental CL system is unable to observe the narrow resonances of the PS particle; in contrast, EEGS can neatly resolve them with observed quality factors $Q \sim 10^4$. We also note that for the silica sphere one observes $Q \sim 100$, and both techniques allow us to resolve the resonances, although EEGS provides a superior spectral precision.

To address this puzzle, we argue that EEGS has the advantage that, under continuous-wave illumination, the spectral width of the laser can be made much narrower than the resonance, so that the electrons interact with a strongly enhanced field. For large quality factors Q such as those of the PS sphere, the CL spectrometer is eventually unable to resolve the spectral shape. Even if an ultraprecise spectrometer is used, capable of resolving resonances with large Q, we find it instructive to compare in what follows the total CL signal integrated over the resonance as a way to quantify the number of electron counts in CL relative to EEGS.

For simplicity, we consider a narrow resonance of frequency ω_0 and quality factor Q in the specimen, for which the spectral dependence of the CL far-field amplitude $\mathbf{f}^{\rm CL}_{\hat{\mathbf{r}}}(\omega)$ in Eq. (S2) can generally be approximated as a Lorentzian, such that

$$\left|\mathbf{f}^{\rm CL}_{\hat{\mathbf{r}}}(\omega)\right|^2 \approx \frac{\left|\mathbf{f}^{\rm CL}_{\hat{\mathbf{r}}}(\omega_0)\right|^2}{4Q^2(\omega/\omega_0-1)^2+1}. \tag{S17}$$

Now, the angle- and frequency-dependent photon emission probability in CL is given by [2] $\Gamma(\hat{\mathbf{r}},\omega) = (c/4\pi\hbar\omega)\left|\mathbf{f}^{\rm CL}_{\hat{\mathbf{r}}}(\omega)\right|^2$, so that the total probability (i.e., integrated over emission angles and frequencies within the resonance peak) reduces to

$$\Gamma_{\rm CL} = \int d^2\hat{\mathbf{r}} \int_{\rm peak} d\omega \left|\mathbf{f}^{\rm CL}_{\hat{\mathbf{r}}}(\omega)\right|^2 \approx \frac{c}{8Q\hbar}\int d^2\hat{\mathbf{r}}\left|\mathbf{f}^{\rm CL}_{\hat{\mathbf{r}}}(\omega_0)\right|^2. \tag{S18}$$

Likewise, from the EEGS probability $\Gamma_{\rm EEGS}(\omega) = |\beta_{\rm EGGS}(\omega)|^2$, using Eq. (S2) for plane-wave illumination, we find

$$\Gamma_{\rm EEGS} = \frac{c^4}{8\pi\hbar^2\omega_0^4}\left|E^{\rm ext}\right|^2 \int d^2\hat{\mathbf{r}}\left|\mathbf{f}^{\rm CL}_{\hat{\mathbf{r}}}(\omega_0)\right|^2, \tag{S19}$$

where, for the sake of our argument, we assume resonant illumination ($\omega = \omega_0$) and introduce an average over orientations of the light field $\mathbf{E}^{\rm ext}$ and the directions $\hat{\mathbf{r}}$ from which light is coming (i.e., we integrate over directions $\hat{\mathbf{r}}$, divide by the full 4π solid angle of the sphere, and also divide by a factor of 2 to account for the average over light polarizations). We have now quantities that can directly be compared to find the dimensionless quantity

$$\mathcal{R}^{\rm EEGS}_{\rm CL} = \frac{Qc^3\left|E^{\rm ext}\right|^2}{\pi\hbar\omega_0^4} = \frac{2Qc^2I^{\rm ext}}{\hbar\omega_0^4}, \tag{S20}$$

which is the ratio of EEGS-to-CL counts normalized per incident electron. Here, we use the relation $I^{\rm ext} = c\left|E^{\rm ext}\right|^2/2\pi$ between the incident light electric field amplitude and the light intensity. For $\hbar\omega_0 = 2\,$eV, this expression can be written as

$$\mathcal{R}_{\rm CL}^{\rm EEGS} = Q\,\frac{I^{\rm ext}}{5\times10^{10}\,{\rm W/m^2}}. \tag{S21}$$

Then, for a plausible light intensity of $10^8\,{\rm W/m^2}$ in our experiment, the ratio with $Q = 200$ (silica sphere) is $\mathcal{R}_{\rm CL}^{\rm EEGS} = 0.4$, and therefore, both CL and EEGS render comparable signals. In contrast, with $Q = 10^4$ (PS sphere), the ratio becomes $\mathcal{R}_{\rm CL}^{\rm EEGS} = 20$, so that EEGS is much stronger and capable of resolving the resonances, whereas the CL signal is too weak. Finally, we note that the actual ratio $\mathcal{R}_{\rm CL}^{\rm EEGS} = 0.4$ should depend on the specific illumination directions, and also on the effect of the mirror.

References

[1] P. Das, J. D. Blazit, M. Tencé, L. F. Zagonel, Y. Auad, Y. H. Lee, X. Y. Ling, A. Losquin, O. S. C. Colliex, F. J. García de Abajo, et al., Ultramicroscopy **203**, 44 (2019).

[2] F. J. García de Abajo, Rev. Mod. Phys. **82**, 209 (2010).

[3] F. J. García de Abajo and V. Di Giulio, ACS Photonics **8**, 945 (2021).

[4] F. J. García de Abajo, Phys. Rev. B **60**, 6086 (1999).

[5] A. Messiah, *Quantum Mechanics* (North-Holland, New York, 1966).

[6] F. J. García de Abajo, Phys. Rev. E **61**, 5743 (2000).

[7] A. Winther and K. Alder, Nucl. Phys. A **319**, 518 (1979).

[8] M. Abramowitz and I. A. Stegun, *Handbook of Mathematical Functions* (Dover, New York, 1972).

[9] E. D. Palik, *Handbook of Optical Constants of Solids* (Academic Press, San Diego, 1985).

[10] G. Mie, Ann. Phys. (Leipzig) **330**, 377 (1908).
